# Efficacy of adjuvant chemotherapy in patients with late-onset stage II/III colon cancer: a retrospective cohort study

**DOI:** 10.1515/med-2026-1442

**Published:** 2026-07-06

**Authors:** Peng Song, Yongjie Zhao, Wencong Tian, Jia Zhao, Yanhong Liu, Chuntao Wang, Lei Cao

**Affiliations:** Department of General Surgery, Tianjin Union Medical Center, The First Affiliated Hospital of Nankai University, Tianjin, China; Tianjin Key Laboratory of General Surgery in Construction, Tianjin Union Medical Center, Tianjin, China

**Keywords:** 50 years and older, chemotherapy, survival, stage II/III, colon cancer

## Abstract

**Objectives:**

Studies providing more evidence to guide adjuvant chemotherapy decisions in colon cancer patients aged 50 years and older are expected.

**Methods:**

Eligible patients were recruited from the Surveillance, Epidemiology and End Results (SEER) database between 2010 and 2015. The cancer-specific survival (CSS) rate was analyzed using the Kaplan–Meier method, and comparisons of survival difference between different subgroups were performed using the log-rank test. Multivariate Cox proportional hazards regression models were built to assess hazard ratios (HRs) of different variables with 95 % confidence intervals (95 % CIs).

**Results:**

In stage II colon cancer patients aged 50 years or older, the Kaplan–Meier survival analysis showed that the 5-year CSS rates of chemotherapy and no chemotherapy groups were 83.5 % and 85.6 %, respectively (p=0.0047). Patients with chemotherapy receipt were associated with a higher cancer-specific mortality rate (HR=1.134, 95 % CI: 1.027–1.252, p=0.013); however, further subgroup analyses indicated that this finding is highly likely driven by unmeasured confounding and selection bias rather than direct treatment-related harm. For stage III patients of the same age group, 5-year CSS was 74.2 % (chemotherapy) vs. 56.4 % (no chemotherapy, p<0.0001), and chemotherapy was linked to a 49.3 % lower cancer-specific mortality (HR=0.507, 95 % CI: 0.482–0.534, p<0.001).

**Conclusions:**

Adjuvant chemotherapy should be considered during the treatment of stage III colon cancer patients aged 50 years or older. For stage II patients, a generalized survival advantage from chemotherapy was not observed, which highlights the profound impact of selection bias in observational data and strongly emphasizes the necessity of precise, individualized risk stratification before administering treatment.

## Introduction

Colon cancer is the most common gastrointestinal malignancy worldwide. In 2018, approximately 1.09 million new cases were diagnosed, which accounted for 6.1 % of the total number of new cases [1]. Based on the age at diagnosis, colon cancer (CC) can be classified into early-onset (EO) colon cancer, diagnosed before the age of 50, and late-onset (LO) colon cancer, diagnosed at the age of 50 or later [2]. Although “late-onset” in the context of traditional geriatric oncology is often operationally defined as aged 65 or 70 years, the epidemiological classification of colorectal cancer increasingly dichotomizes the disease at the age of 50. This threshold aligns with established average-risk screening paradigms and effectively distinguishes conventional cases from the clinically and biologically distinct early-onset demographic. Regarding the change in incidence rate, most data indicate that it has decreased among patients aged over 50 but remained stable in developed countries [3], 4]. Currently, surgery, radiotherapy, and systemic chemotherapy are the standard of care for colon cancer patients. Approximately 72 % of newly diagnosed colon cancer patients present with local or regional disease [5], offering an opportunity for curative-intent treatment.

Adjuvant chemotherapy following surgical resection of colon cancer can not only eliminate micrometastatic disease and consolidate the surgical effect but also enhance overall and disease-free survival [6]. Evidence from randomized controlled trials (RCTs) has firmly established that adjuvant chemotherapy significantly improves survival rates among stage III colon cancer patients [7], 8]. Consequently, adjuvant chemotherapy has become the standard of care for stage III colon cancer. However, for stage II colon cancer patients who have undergone complete surgical resection, adjuvant chemotherapy remains a challenging decision. This is mainly due to the fact that, in stage II, surgery alone can achieve a relatively high cure rate, while adjuvant chemotherapy with fluoropyrimidines offers only marginal benefits (less than 5 %) [9]. In fact, various clinical trials and meta-analyses have reported inconsistent findings regarding the actual benefits of adjuvant therapy in stage II colon cancer. The rationale behind this study stems from the ongoing controversy surrounding the actual survival benefits of adjuvant chemotherapy in stage II late-onset colon cancer, primarily due to the lack of precise risk stratification in clinical practice. We hypothesized that the seemingly poor outcomes of stage II patients receiving chemotherapy observed in previous observational cohorts are predominantly driven by severe selection bias rather than the direct harmful effects of the treatment. Therefore, the present study aimed to answer the following research question: What is the real-world efficacy of adjuvant chemotherapy in patients aged ≥50 years with stage II and stage III colon cancer, and how does selection bias influence the observed survival outcomes in the stage II population?

## Materials and methods

### Data source and patient selection

**Figure 1:**
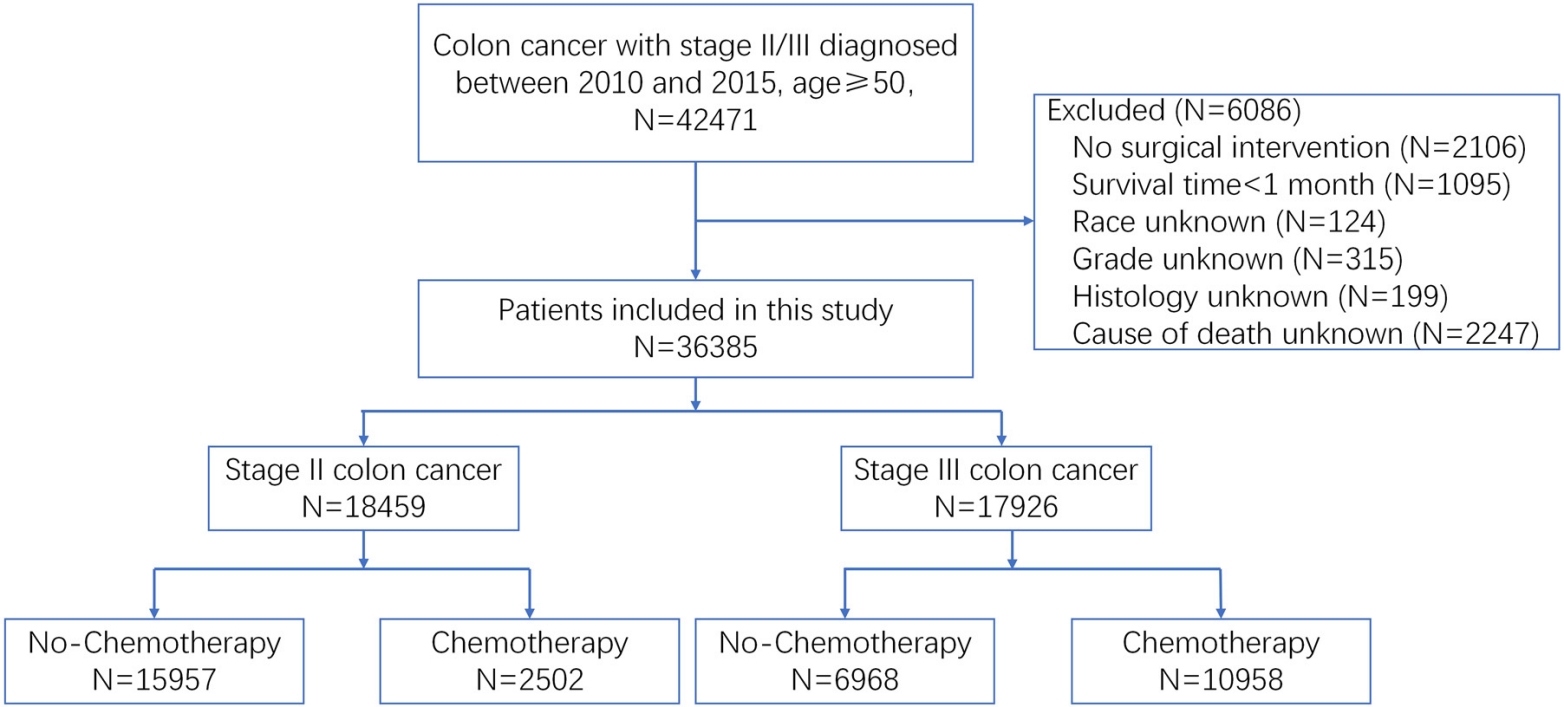
Flow diagram of eligible patients selected from the SEER Database.

In this retrospective analysis, we obtained data from the Surveillance, Epidemiology and End Results (SEER) database between 2010 and 2015, which was maintained by the National Cancer Institute and covered basic information for ∼28 % of the US population [10]. We obtained permission to download data from the SEER Database. All information has been anonymized and cannot be used to identify individual participants, so obtaining informed consent from patients is not required. The data extraction date was October 6, 2024; the time frame for conducting the statistical analysis was from October to November 2024. Inclusion Criteria: (1) Year of diagnosis between 2010 and 2015; (2) Select colon excluding rectum by site code ICD-O-3; (3) Aged≥50 years; (4) Colon cancer was the only primary malignancy; (5) AJCC stage II–III. Exclusion criteria: (1) No surgical intervention; (2) Follow-up duration less than 1 month; (3) The basic information of the patient is incomplete. The AJCC 7th edition was used to determine clinicopathological staging [11]. The selection criteria and screening process are illustrated in Figure 1.

This was a retrospective cohort study based on the SEER database. No prospective sample size calculation or power analysis was performed, since the sample size was determined by the inclusion and exclusion criteria applied to the database.

### Study variables

The variables analyzed in this study included gender, race, grade, histological type, stage, chemotherapy status, survival status, and survival months. These variables were categorized as follows: race was classified into White, Black, and Other (including Asian or Pacific Islander); tumor grade was grouped into well/moderately differentiated (Grade I–II) and poorly differentiated/undifferentiated (Grade III–IV); histological type was categorized as adenocarcinoma, signet ring cell carcinoma, or mucinous adenocarcinoma; regarding chemotherapy, status is recorded in the SEER database as “Yes” or “No/Unknown”. In accordance with standard practices for SEER data analyses, patients coded as “No/Unknown” were grouped together and defined as the “No-Chemotherapy” group in our study.

The cancer-specific survival (CSS) was the primary survival outcome, defined as the time from diagnosis to death attributable to colon cancer. Deaths from other causes were treated as censored observations in the CSS analysis.

### Statistical analysis

Descriptive statistics of patient characteristics were summarized. Chi‐square analysis was used to analyze and evaluate the categorical data. The Kaplan–Meier curve was used to estimate the CSS in different groups, and the differences between the curves were analyzed by log‐rank test. Multivariate Cox proportional hazard regression was used to determine independent prognostic factors, and a hazard ratio (HR) and corresponding 95 % confidence interval (CI) were calculated. The proportional hazards assumption was carefully assessed and verified using Schoenfeld residuals, and no significant violations were observed. The statistical analyses were carried out using SPSS Statistical Software version 26.0 (IBM Corp., USA) and the R package “survival”, where a two-sided p<0.05 was considered statistically significant.

## Results

### Demographic and clinical characteristics

A total of 36,385 patients aged 50 years or older diagnosed with stage II/III colon cancer between 2010 and 2015 were recruited from the SEER database, including 18,459 patients (50.7 %) with stage II disease, and 17,926 patients (49.3 %) with stage III disease, 17,332 males (47.6 %) and 19,053 females (52.4 %), most of them were white (78.7 %). In colon patients with known tumor grade, grade I/II (28,386, 78.0 %) patients were far more than grade III/IV (7,999, 22.0 %) patients; in colon patients with known histology, patients with adenocarcinoma (32,273, 88.7 %) were far more than patients with mucinous adenocarcinoma/signet ring cell carcinoma (4,112, 11.3 %). The average follow-up time for all patients was 94.5 months (range, 1–119 months).

Table 1 summarized the demographic and clinicopathological characteristics of stage II/III colon cancer patients by the receipt of chemotherapy. There was a significant difference in gender, race, tumor grade, histology and TNM stage among the patients. Patients in the Chemo group presented with higher percentage of male (49.9 vs. 46.3 %), higher black population (12.6 vs. 10.5 %), higher histologic grade (III and IV; 25.3 vs. 20.0 %), higher percentage of stage III (81.4 vs. 30.4 %), and more Signet ring cell carcinoma (1.5 vs. 1.0 %) (all p<0.001) compared with those in the No-Chemo group. Notably, these baseline characteristics reveal a substantial clinical imbalance between the two cohorts, clearly indicating that patients receiving chemotherapy inherently possessed significantly higher-risk tumor features (e.g., more advanced stage, higher tumor grade, and more aggressive histology) at baseline compared to those who did not receive chemotherapy.

**Table 1:** Patient characteristics of stage II/III colon cancer.

Variables	Chemotherapy		p-Value
	No (N=22,925)	Yes (N=13,460)	
Gender			<0.001
Male	10,619 (46.3)	6,713 (49.9)	
Female	12,306 (53.7)	6,747 (50.1)	
Race			<0.001
White	18,308 (79.9)	10,338 (76.8)	
Black	2,403 (10.5)	1,699 (12.6)	
Other	2,214 (9.6)	1,423 (10.6)	
Grade			<0.001
Grade I/II	18,329 (80.0)	10,057 (74.7)	
Grade III/IV	4,596 (20.0)	3,403 (25.3)	
Histology			<0.001
Adenocarcinoma	20,314 (88.6)	11,959 (88.8)	
Signet ring cell carcinoma	230 (1.0)	198 (1.5)	
Mucinous adenocarcinoma	2,381 (10.4)	1,303 (9.7)	
Stage			<0.001
II	15,957 (69.6)	2,502 (18.6)	
III	6,968 (30.4)	10,958 (81.4)	

Table 2 summarizes the demographic and clinicopathological characteristics of colon cancer patients by different stages. There was a significant difference in race, tumor grade, histology and adjuvant chemotherapy among the patients. Statistically significant differences (p<0.001) were noted, between the stage III group and the stage II group, in race (11.2 vs. 8.8 % other), histologic grade (27.2 vs. 16.9 % III/IV), histology (1.8 vs. 0.6 % signet ring cell carcinoma), adjuvant chemotherapy (61.1 vs. 13.6 % yes).

**Table 2:** Clinicopathological characteristics of colon cancer patients by stage group.

Variables	Stage		p-Value
	II (N=18,459)	III (N=17,926)	
Gender			0.770
Male	8,779 (47.6)	8,553 (47.7)	
Female	9,680 (52.4)	9,373 (52.3)	
Race			<0.001
White	14,909 (80.8)	13,737 (76.6)	
Black	1,918 (10.4)	2,184 (12.2)	
Other	1,632 (8.8)	2,005 (11.2)	
Grade			<0.001
Grade I/II	15,334 (83.1)	13,052 (72.8)	
Grade III/IV	3,125 (16.9)	4,874 (27.2)	
Histology			<0.001
Adenocarcinoma	16,358 (88.6)	15,915 (88.8)	
Signet ring cell carcinoma	105 (0.6)	323 (1.8)	
Mucinous adenocarcinoma	1,996 (10.8)	1,688 (9.4)	
Chemotherapy			<0.001
No	15,957 (86.4)	6,968 (38.9)	
Yes	2,502 (13.6)	10,958 (61.1)	

### The effect of adjuvant chemotherapy in stage II colon cancer patients aged 50 years or older

**Figure 2:**
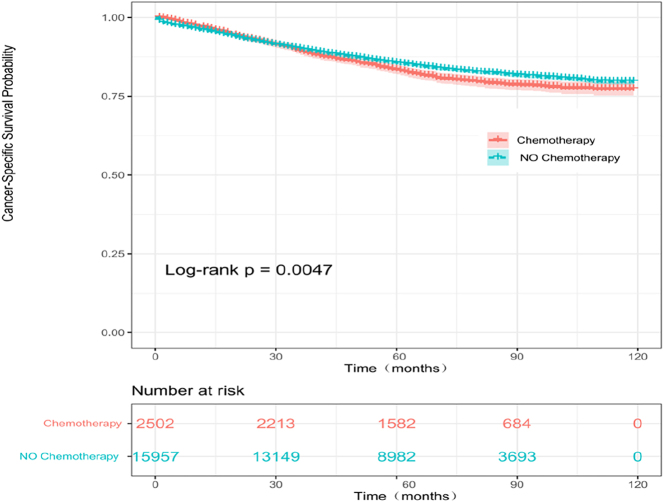
Kaplan–Meier curves of survival probability in stage II colon cancer patients with and without chemotherapy.

In stage II colon cancer patients aged 50 years or older, the Kaplan–Meier survival analysis showed that the 5-year CSS rates of chemotherapy and no chemotherapy groups were 83.5 % and 85.6 %, respectively (p=0.0047), suggesting that the receipt of chemotherapy was not associated with a survival advantage in the stage II cohort (Figure 2).

In Table 3, univariate and multivariate Cox analyses were conducted to evaluate the prognostic characteristics in colon cancer patients of stage II. The univariate analysis produced three variables that were then included in the multivariate analysis. For the whole cohort, race, histologic grade, and the receipt of chemotherapy were independent prognostic determinants of CSS in stage II colon cancer patients aged 50 years or older. Apart from this, it was found that the receipt of chemotherapy had significantly 13.4 % increased risk of cancer specific mortality (HR=1.134, 95 % CI=1.027–1.252, p=0.013).

**Table 3:** Cox regression analyses of prognostic factors for CSS in stage II colon cancer.

Variables	Univariate analyses			Multivariate analyses		
	HR	95 % CI	p-Value	HR	95 % CI	p-Value
Gender			0.532			
Male	Reference					
Female	1.024	0.951–1.101				
Race			0.001			0.001
White	Reference			Reference		
Black	1.142	1.020–1.279		1.151	1.028–1.289	0.015
Other	0.819	0.712–0.942		0.828	0.720–0.952	0.008
Grade			<0.001			0.001
Grade I/II	Reference			Reference		
Grade III/IV	1.209	1.101–1.326		1.200	1.094–1.318	
Histology			0.695			
Adenocarcinoma	Reference					
Signet ring cell carcinoma	1.205	0.767–1.892				
Mucinous adenocarcinoma	1.018	0.905–1.144				
Chemotherapy			0.005			0.013
No	Reference			Reference		
Yes	1.153	1.044–1.273		1.134	1.027–1.252	

### Subgroup analysis of adjuvant chemotherapy in stage II colon cancer

**Figure 3:**
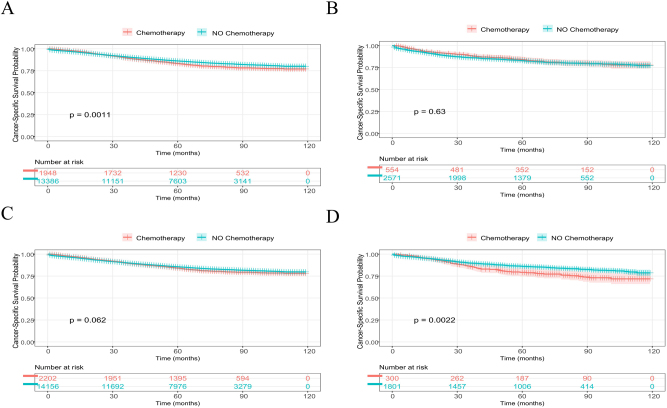
Kaplan–Meier curves of cancer-specific survival probability for stage II colon cancer subgroups with and without adjuvant chemotherapy. Stratified survival analyses were performed for specific high-risk subgroups to compare outcomes between patients who received chemotherapy (red) and those who did not (blue/green). (A) Patients with well/moderately differentiated tumors (Grade I/II). (B) Patients with poorly differentiated/undifferentiated tumors (Grade III/IV). (C) Patients with conventional adenocarcinoma. (D) patients with signet ring cell or mucinous adenocarcinoma.

To further investigate the effect of chemotherapy in specific high-risk subgroups of stage II patients, we performed unadjusted Kaplan–Meier analyses and adjusted multivariate Cox regression models (Table 4). Consistent with the overall stage II cohort, unadjusted Kaplan–Meier curves suggested no benefit or even worse survival for chemotherapy groups across the examined subgroups (Figure 3). After adjusting for potential confounders in the multivariate Cox regression models (using the chemotherapy group as the reference), the results remained consistent. Not receiving chemotherapy was independently associated with a significantly decreased risk of cancer-specific mortality in patients with well/moderately differentiated tumors (Grade I/II; HR=0.847, 95 % CI: 0.758–0.947, p=0.004) and those with signet ring cell or mucinous adenocarcinoma (HR=0.657, 95 % CI: 0.505–0.862, p=0.002). No significant survival differences were observed between the two groups in patients with poorly differentiated/undifferentiated tumors (Grade III/IV; HR=1.060, 95 % CI: 0.854–1.316, p=0.597) or conventional adenocarcinoma (HR=0.932, 95 % CI: 0.837–1.038, p=0.199).

**Table 4:** Effect of adjuvant chemotherapy on CSS in subgroups of stage II colon cancer.

Subgroups	Chemo	No-chemo	HR (no-chemo vs. chemo)	95 % CI	p-Value
Grade I/II	1,948	13,386	0.847	0.758–0.947	0.004
Grade III/IV	554	2,571	1.060	0.854–1.316	0.597
Adenocarcinoma	2,202	14,156	0.932	0.837–1.038	0.199
Signet ring/mucinous	300	1,801	0.657	0.505–0.862	0.002

### The effect of adjuvant chemotherapy in stage III colon cancer patients aged 50 years or older

**Figure 4:**
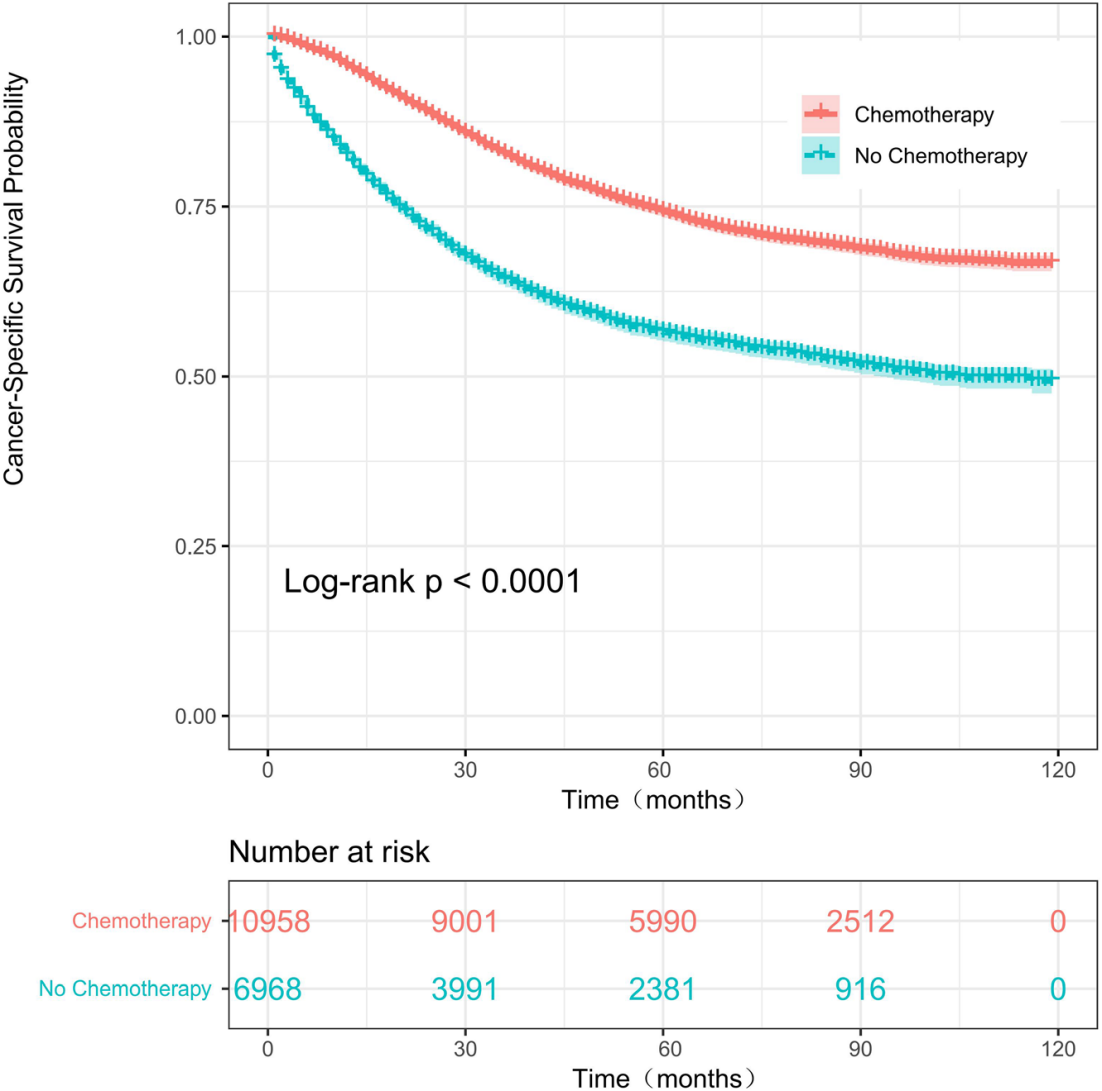
Kaplan–Meier curves of survival probability in stage III colon cancer patients with and without chemotherapy.

In stage III colon cancer patients aged 50 years or older, the Kaplan–Meier survival analysis showed that the 5-year CSS rates of chemotherapy and no chemotherapy groups were 74.2 and 56.4 %, respectively (p<0.0001), demonstrating a pronounced survival benefit associated with adjuvant chemotherapy in the stage III cohort (Figure 4).

In Table 5, univariate and multivariate Cox analyses were conducted to evaluate the prognostic characteristics in colon cancer patients of stage III. The univariate analysis produced four variables that were then included in the multivariate analysis and the variable of gender was excluded. For the whole cohort, race, histologic grade, histology, and the receipt of chemotherapy were independent prognostic determinants of CSS in stage III colon cancer patients aged 50 years or older. In stage III cancer patients aged 50 years or older, the receipt of chemotherapy had significantly 49.3 % decreased risk of cancer-specific mortality (HR=0.507, 95 % CI=0.482–0.534, p<0.001).

**Table 5:** Cox regression analyses of prognostic factors for CSS in stage III colon cancer.

Variables	Univariate analyses			Multivariate analyses		
	HR	95 % CI	p*-*Value	HR	95% CI	p-Value
Gender			0.023			
Male	Reference					
Female	1.061	1.008–1.117				
Race			<0.001			<0.001
White	Reference			Reference		
Black	1.040	0.963–1.123		1.085	1.005–1.172	0.038
Other	0.829	0.760–0.904		0.850	0.779–0.927	<0.001
Grade			<0.001			<0.001
Grade I/II	Reference			Reference		
Grade III/IV	1.749	1.658–1.845		1.656	1.567–1.750	
Histology			<0.001			<0.001
Adenocarcinoma	Reference			Reference		
Signet ring cell carcinoma	2.743	2.377–3.167		1.872	1.615–2.169	<0.001
Mucinous adenocarcinoma	1.298	1.196–1.410		1.261	1.162–1.370	<0.001
Chemotherapy			<0.001			<0.001
No	Reference			Reference		
Yes	0.504	0.479–0.531		0.507	0.482–0.534	

## Discussion

In the present study, we found that adjuvant chemotherapy confers a substantial and independent survival benefit in late-onset stage III colon cancer patients, significantly reducing the risk of cancer-specific mortality. For the stage II cohort, however, chemotherapy did not demonstrate a generalized survival advantage, which is highly likely attributable to unmeasured confounding and selection bias rather than direct treatment-related harm. Colorectal cancer (CRC) is the third most common cancer worldwide and the second leading cause of cancer-related mortality [12]. The full implementation and popularization of CRC screening strategies in recent years have led to a significant decrease in morbidity and mortality rates in the USA and European countries [13], 14]. However, it continues to rise in developing countries. Population aging and changes in lifestyle are the core driving factors. In 2022, there were approximately 1.9 million new cases of colorectal cancer globally, with 904,000 deaths. People aged 50 and above accounted for more than 90 % of the new cases. In clinical practice, patients with late-onset colorectal cancer exhibit lower adjuvant chemotherapy adherence compared to their early-onset counterparts [15].

Radical resection is a cornerstone of curative intent treatment for colorectal cancer, aiming to completely excise the tumor, adjacent potentially invaded tissues, and regional lymph nodes to achieve oncological cure. Over the past few decades, there have been significant improvements in the treatment of patients with colonic and rectal cancers. Of these, arguably the most important for the surgeon was the advent of total mesorectal excision (TME) by Heald and Ryall [16]. Based on the TME experience, the group from Erlangen in Germany have advocated for CME in conjunction with CVL for colon cancer [17], 18]. The Erlangen group has also shown that CME improves 5-year survival rates and locoregional recurrence rates for their patients. The improvements are not as marked as with TME, but the improvement in local recurrence, from 6.5 to 3.6 %, and in 5-year survival, from 82.1 to 89.1 %, are striking [19]. The outcomes of the Erlangen group have since been replicated by others, suggesting that high vascular ligation shows oncological benefit in both local recurrence and 5-year mortality rates. The apparent improved outcomes with CME are yet to be confirmed with a formal RCT.

Postoperative chemotherapy – initially fluoropyrimidines and more recently combinations with oxaliplatin – has reduced the risk of tumor recurrence and improved survival for patients with resected colon cancer. Although universally recommended for patients with stage III disease, there is no consensus about the survival benefit of postoperative chemotherapy in stage II colon cancer. Common chemotherapeutic agents play a pivotal role in the treatment of late-onset colorectal cancer. 5-fluorouracil (5-FU), a cornerstone of CRC chemotherapy, exerts antitumor effects by inhibiting thymidylate synthase, thereby disrupting DNA synthesis [20]. Oxaliplatin, a platinum-based agent, induces DNA cross-links to suppress DNA replication and transcription, demonstrating potent cytotoxicity [21]. Clearly, a great deal has changed in medical and surgical therapy for resectable colon cancer since the benefit of postoperative fluoropyrimidine-based therapy for patients with stage III and selected stage II disease was established in the early 1990s.

Hence, we conducted this retrospective analysis aiming to provide more evidence to guide adjuvant chemotherapy decisions in late-onset colorectal cancer patients.

Our findings demonstrated that among patients with stage III late-onset colon cancer who received adjuvant chemotherapy, the 5-year CSS rate increased from 56.4 to 74.2 %, with a significant 49.3 % reduction in the risk of cancer-specific mortality. These results are consistent with current clinical consensus and further validate the therapeutic necessity of adjuvant chemotherapy for patients with stage III late-onset colon cancer. However, the findings in stage II patients were inconsistent with expectations: the 5-year CSS rate in those who received chemotherapy (83.5 %) was significantly lower than that in those who did not (85.6 %), and chemotherapy was associated with a 13.4 % increase in the risk of cancer-specific mortality. This “unexpected result” requires in-depth interpretation in combination with recent evidence-based medicine data and the limitations of the study design.

To address the hypothesis that specific subsets of stage II patients might inherently benefit from adjuvant chemotherapy, we conducted exploratory subgroup analyses stratified by tumor grade and histology. Interestingly, both unadjusted survival curves and multivariate Cox regression models failed to demonstrate a survival benefit for chemotherapy, even in traditional high-risk subgroups such as poorly differentiated tumors or signet ring cell/mucinous adenocarcinoma. In fact, not receiving chemotherapy was independently associated with a significantly lower risk of mortality in certain subgroups (e.g., HR=0.847 for Grade I/II and HR=0.657 for signet ring cell/mucinous adenocarcinoma). Rather than proving that chemotherapy is biologically harmful, these persistent negative results powerfully underscore the profound impact of selection bias inherent in retrospective database analyses. In real-world clinical practice, stage II patients who are ultimately prescribed chemotherapy – even within the same histological or grading risk category – likely possess severe, unmeasured high-risk clinical characteristics (e.g., bowel obstruction, perforation, perineural invasion, or inadequate lymph node yield) that predispose them to worse intrinsic outcomes. Furthermore, the lack of molecular data, such as mismatch repair (MMR) status, in the SEER database limits our ability to identify patients who might genuinely benefit, as deficient MMR (dMMR) stage II tumors are known to lack benefit from fluorouracil-based therapy. These findings reiterate the critical need for comprehensive pathological and molecular risk stratification to guide individualized treatment decisions in stage II colon cancer.

Pooled analyses from studies such as the IDEA Collaborative Group series and the TOSCA trial have indicated that even for patients with high-risk stage II colon cancer, the absolute benefit of adjuvant chemotherapy remains very limited and largely depends on accurate risk stratification. For instance, patients with stage II colon cancer exhibiting the molecular phenotype of deficient mismatch repair/microsatellite instability-high (dMMR/MSI-H) not only fail to benefit from fluorouracil-based monochemotherapy but may even experience treatment-related adverse outcomes [22]. The results of this study further emphasize the importance of avoiding overtreatment in patients with stage II colon cancer and indirectly suggest that without precise risk stratification, the generalized administration of chemotherapy to all stage II patients may fail to deliver effective treatment to those who would truly benefit, while exposing some low-risk patients to unnecessary treatment-related toxicities. Future clinical decisions should rely more heavily on comprehensive pathological reports and molecular marker detection results. Looking forward, the precise management of colorectal cancer and the boundary of adjuvant chemotherapy benefits will increasingly rely on novel theranostic strategies and a deeper understanding of the tumor microenvironment. Recent advances have highlighted that the crosstalk between host immunity, systemic inflammation, and gut microbiota – including the pathogenic roles of specific bacterial extracellular vesicles (e.g., from *Streptococcus anginosus*) in modulating immune cells – can significantly impact disease progression and therapeutic efficacy across various malignancies and autoimmune conditions [23], [24], [25], [26], [27]. Furthermore, the development of intelligent, targeted nanoplatforms and cellular immune theranostic approaches are currently revolutionizing the ways we overcome drug resistance and enhance localized anti-tumor immunity [28], [29], [30], [31]. Integrating these multidimensional biomarkers (e.g., specific gut microbiome signatures, extracellular vesicles) and novel nanomedicine delivery systems into the clinical evaluation of late-onset colon cancer will be pivotal for achieving true precision oncology in the future.

The core value of this study lies in the following: based on a large-sample cohort (36,385 cases) of patients with stage II/III late-onset (≥50 years) colon cancer, it is the first study to clearly confirm the significant survival benefit of adjuvant chemotherapy in stage III patients, thereby providing real-world evidence support for the clinical recommendation that “adjuvant chemotherapy should be actively recommended for patients with stage III late-onset colon cancer”. Meanwhile, it suggests that chemotherapy decisions for patients with stage II late-onset colon cancer require “individualized assessment” rather than a one-size-fits-all approach. Although this study adjusted for some available variables through multivariate analysis, the SEER database lacks key information such as patients’ performance status (PS score), specific comorbidities, surgical complications, molecular pathological features (e.g., microsatellite instability status), and considerations in doctor-patient treatment decision-making. In clinical practice, stage II patients who receive adjuvant chemotherapy may inherently belong to the high-risk group, presenting with conditions such as tumor obstruction, perforation, lymphovascular invasion, perineural invasion, or fewer than 12 lymph nodes examined. Therefore, the survival difference observed in this study is more likely to reflect the selection bias of “patients with more severe conditions receiving more intensive treatment” rather than the harmful effects of chemotherapy itself. Additionally, the possibility of data coding errors cannot be completely ruled out.

This study has the following limitations. First, the conclusions are based on a retrospective analysis of the SEER database, which is inherently susceptible to potential selection bias. Second, while AJCC 7th edition substaging (IIA, IIB, IIC, IIIA, IIIB, IIIC) is available in the SEER database, this study only adopted the broad stage II/III groupings for analysis and did not perform further analyses based on detailed substages. Third, although the SEER database provides reliable population-level data, the administration of chemotherapy is not randomly assigned. Fourth, the SEER database does not include key individualized information, such as patients’ performance status (ECOG/PS score), specific comorbidities, surgical complications, perineural/lymphovascular invasion, socioeconomic status, and treatment willingness. These unmeasured confounding factors – especially performance status and comorbidities – are likely the primary reasons for the “worse survival outcomes in the chemotherapy group” among stage II patients. Fifth, the SEER database lacks detailed granular information regarding specific chemotherapy regimens (e.g., 5-FU monotherapy vs. oxaliplatin-based combinations like FOLFOX or CAPOX), the number of completed cycles, and overall dose intensity. This critical gap prevents us from assessing chemotherapy efficacy by treatment type or evaluating the impact of dose modifications. Consequently, we cannot rule out the possibility that suboptimal chemotherapy delivery or severe treatment-related toxicities – which might outweigh the marginal survival benefits in older populations – partially explain the lack of a generalized survival advantage observed in our stage II cohort. Future prospective studies and registries incorporating detailed treatment data, including specific regimens, cumulative doses, and toxicity profiles, are urgently needed to fully address these limitations and to accurately define the optimal treatment strategies for late-onset colon cancer.

## Conclusions

Taken together, this study provides robust real-world evidence for administering adjuvant chemotherapy to patients aged ≥ 50 years with stage III colon cancer, supporting the active recommendation of chemotherapy in this population. In contrast, a generalized survival advantage for chemotherapy was not observed in stage II patients. The seemingly inferior outcomes associated with chemotherapy in the stage II cohort, even within specific high-risk histological subgroups, are highly indicative of unmeasured confounding and selection bias – whereby patients with heavier unrecorded clinical burdens or higher baseline risks are preferentially triaged to receive treatment – rather than a direct harmful effect of the chemotherapy itself. These findings highlight the urgency of formulating treatment strategies based on meticulous individualized assessment and precise risk stratification to avoid one-size-fits-all overtreatment. Prospective studies are needed in the future, and greater efforts should be made to encourage the participation of more elderly patients in randomized controlled trials to clarify the actual boundary of adjuvant chemotherapy benefits in stage II colon cancer, particularly in the late-onset population.
